# Rural Versus Urban Genitourinary Cancer Incidence and Mortality in Pennsylvania: 1990–2019

**DOI:** 10.3390/curroncol31120597

**Published:** 2024-12-23

**Authors:** Jonathan Pham, Ahmad N. Alzubaidi, Jay D. Raman, Tullika Garg

**Affiliations:** Department of Urology, Penn State Health Milton S. Hershey Medical Center, Hershey, PA 17033, USA; jpham1@pennstatehealth.psu.edu (J.P.); analzubaidi@gmail.com (A.N.A.); jraman@pennstatehealth.psu.edu (J.D.R.)

**Keywords:** bladder cancer, kidney cancer, prostate cancer, rural health, health disparities, epidemiology, urologic oncology, cancer control

## Abstract

Our aim was to describe the incidence and mortality of genitourinary (GU) cancers in rural and urban Pennsylvania counties. We calculated age-adjusted incidence and mortality rates of GU (prostate, bladder, and kidney) cancers from 1990 to 2019 in the Pennsylvania Cancer Registry. We defined rurality using the Center for Rural Pennsylvania’s population density-based definition. We modeled average annual percent changes (AAPC) in age-adjusted incidence and mortality rates using joinpoint regression. Overall GU cancer incidence decreased in rural and urban counties (AAPC −7.5%, *p* = 0.04 and AAPC −6.6%, *p* = 0.02, respectively). Prostate cancer incidence decreased in rural and urban counties by −10.5% (*p* = 0.02) and −9.1% (*p* = 0.01), respectively. Kidney cancer incidence increased in both rural and urban counties, respectively (AAPC = +11.2, *p* = 0.002 and +9.3%, *p* = 0.01). GU cancer mortality decreased in rural and urban counties (AAPC = −11.6, *p* = 0.047 and AAPC −12.2, *p* = 0.01, respectively). Prostate cancer mortality decreased at similar rates in rural and urban counties (AAPC −15.5, *p* = 0.03 and −15.4, *p* = 0.02, respectively). Kidney cancer mortality decreased in urban (AAPC −6.9% *p* = 0.03) but remained stable in rural counties. Bladder cancer incidence and mortality were unchanged in both types of counties. Over three decades, GU cancer incidence and mortality decreased across Pennsylvania counties.

## 1. Introduction

The burden of genitourinary (GU) cancers including prostate, bladder, and kidney cancer is enormous, accounting for approximately 25% of all cancer survivors in the United States (U.S) [1]. Prostate cancer is the most common cancer diagnosed in U.S. men, bladder cancer is the fourth most common cancer in men and the sixth most common across both sexes and kidney cancer is the ninth most common cancer. Pennsylvania ranks twenty-second among U.S. states in cancer mortality rates, and cancer is the second leading cause of death in the state [2]. Prostate cancer and bladder cancer are projected to be in the top five cancers for new diagnoses in Pennsylvania in 2023, with an anticipated 13,210 and 4270 new cases, respectively [3,4].

Cancer incidence and mortality rates have been decreasing overall in the United States; however, rural areas have seen slower declines in cancer mortality compared to urban areas, which may be in part due to higher death rates from certain cancers including prostate [4,5]. Despite the high incidence and widespread prevalence of GU cancers, few studies have evaluated urban–rural differences in overall and individual GU cancer incidence and mortality. Most existing studies have focused only on prostate cancer and have not included bladder and/or kidney which are also common and contribute significantly to cancer burden in the U.S. The aim of this study was to describe changes in incidence and mortality of genitourinary (GU) cancers in rural and urban counties in the Commonwealth of Pennsylvania over approximately three decades using Pennsylvania Cancer Registry data from 1990 to 2019 [6].

## 2. Materials and Methods

### 2.1. Data

This study utilized the Pennsylvania Cancer Registry (PCR), a statewide system that collects data on all new cancers diagnosed and/or treated in the Commonwealth of Pennsylvania. Since 1985, statewide hospitals, clinics, laboratories, radiation facilities, cancer centers, surgical centers, and physician offices have been reporting data to the PCR. Death certificates and interstate data exchanges also provide data when residents of the Commonwealth of Pennsylvania undergo diagnosis or treatment outside the state. The PCR is a member of the National Program of Cancer Registries administered by the U.S. Centers for Disease Control and meets national standards for data reporting. Data are publicly available for download through the PCR’s Enterprise Data Dissemination Information Exchange (EDDIE, no accession number available for this dataset) [7].

We chose to exclude testicular and penile cancer and focus on prostate cancer, bladder cancer, and kidney cancer as these three GU malignancies are consistently among the top ten most common cancers in the U.S. We defined overall GU cancers as the sum of prostate cancer, bladder cancer, and kidney cancer. We obtained age-adjusted incidence, mortality, and rural/urban status for each disease site from the PCR. The data were obtained in intervals of 5 years. Incidence data were available from 1990 to 2019 and mortality data were available from 2000 to 2019. This study was deemed IRB-exempt as we used a publicly available database containing de-identified data.

### 2.2. Rural Definition

We classified Pennsylvania counties as rural or urban using 2019 county-level census data and the Center for Rural Pennsylvania’s 2010 population density-based definition of rural (284 persons per square mile) [8]. Pennsylvania has 67 counties and, based on the Center for Rural Pennsylvania’s definition, 48 counties were classified as rural (71.6%) and 19 counties were classified as urban (28.4%).

### 2.3. Statistical Analysis

We calculated descriptive statistics of the proportions of overall GU cancer, prostate cancer, bladder cancer, and kidney cancer by 5-year increments and rural/urban status. We utilized the National Cancer Institute’s Joinpoint Regression Program to model annual percent changes (APC) and average annual percent change (AAPC, Joinpoint Regression Program, Version 4.9.1.0, Statistical Methodology and Applications Brant, Surveillance Research Program, National Cancer Institute). This analysis program is publicly available for download from the National Cancer Institute’s website (https://surveillance.cancer.gov/joinpoint/, accessed 17 December 2024). The National Cancer Institute uses standardized methods for joinpoint regression. Specifically, the joinpoint program fits a piecewise linear regression model to longitudinal trend data over a given time interval and employs a Monte Carlo hypothesis testing procedure to determine if additional joinpoints need to be added to the model [9]. Inputs included standard errors for age-adjusted incidence rates. We used year as the dependent variable. We fitted separate piecewise linear models for rural and urban incidence and mortality using rural/urban status as the “by” variable. AAPCs provide a summary measure of trends over a specified time interval by computing a weighted average of APCs based on weights equal to the length of each APC time interval. Using joinpoint regression, we calculated AAPCs to describe the average APCs over multiple years for overall GU cancer, prostate cancer, bladder cancer, and kidney cancer for age-adjusted incidence (1990–2019) and for mortality (2000–2019).

## 3. Results

**Figure 1:**
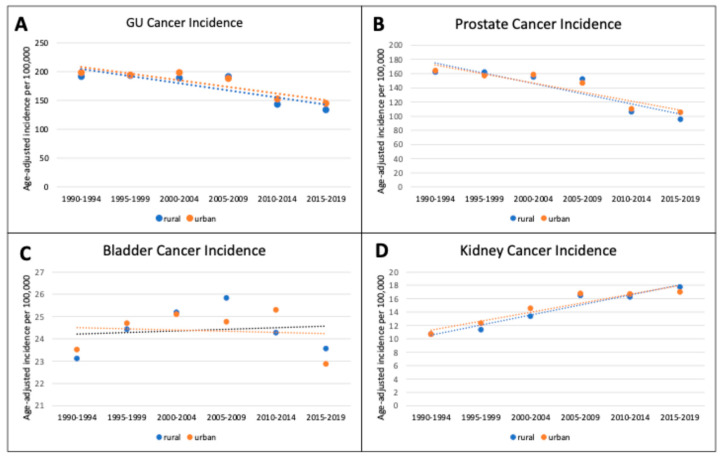
Age-adjusted genitourinary cancer incidence in rural and urban Pennsylvanian counties (1990–2019): (**A**) Overall genitourinary (GU) cancer incidence; (**B**) prostate cancer incidence; (**C**) bladder cancer incidence; (**D**) kidney cancer incidence. Age-adjusted incidence per 100,000 for each time interval with joinpoint regression line.

**Figure 2:**
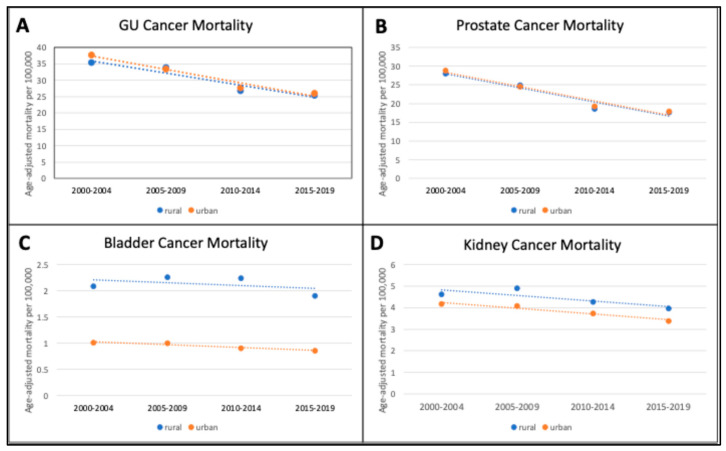
Age-adjusted genitourinary cancer mortality in rural and urban Pennsylvanian counties (1990–2019): (**A**) Overall genitourinary (GU) cancer mortality; (**B**) prostate cancer mortality; (**C**) bladder cancer mortality; (**D**) kidney cancer mortality. Age-adjusted mortality per 100,000 for each time interval with joinpoint regression line.

From 1990 to 2019, there were a total of 462,900 new GU cancer cases diagnosed in Pennsylvania. Prostate cancer contributed the largest proportion (62%), followed by bladder cancer (24%), and kidney cancer (14%). The proportions of new diagnoses of overall GU cancers, prostate cancer, bladder cancer, and kidney cancer remained stable in both urban and rural counties (Table 1). In rural counties, there was a significant 30% decrease in age-adjusted GU cancer incidence from 191.1 cases per 100,000 to 133.6 cases per 100,000 (AAPC −7.5, *p* = 0.04, Figure 1A). Similarly, in urban counties, GU cancer incidence significantly decreased from 198.7 to 145.3 cases per 100,000 (26.9% decrease, AAPC −6.6, *p* = 0.02). From 2000 to 2019, there was a non-significant decrease in age-adjusted GU cancer mortality in rural counties from 35.4 to 25.4 cases per 100,000 (28.0% decrease, AAPC = −11.6, *p* = 0.05). Age-adjusted GU cancer mortality also decreased in urban counties from 37.8 to 26.0 cases per 100,000 (31.2% decrease, AAPC −12.2, *p* = 0.01, Figure 2A).

For prostate cancer, approximately three-quarters of new cases were in urban counties (71.7%) versus rural counties (28.3%, Table 1). From 1990 to 2019, age-adjusted prostate cancer incidence decreased in rural counties by 70% from 162.2 to 95.7 (AAPC −10.5, *p* = 0.02) and in urban counties by 56% from 164.7 to 105.4 cases per 100,000 (AAPC −9.1, *p* = 0.01, Figure 1B). Prostate cancer mortality decreased at similar rates for rural (37.1% decrease, AAPC −15.5, *p* = 0.03) and urban counties (37.9% decrease, AAPC −15.4, *p* = 0.02, Figure 2B).

Urban counties had the largest proportion of new bladder cancer diagnoses over the study period (70.7%, Table 1). Unlike prostate cancer, the age-adjusted incidence of bladder cancer remained stable for both rural and urban counties (*p* = 0.90 versus *p* = 0.75, Figure 1C). Age-adjusted mortality for bladder cancer also remained stable for both rural and urban counties over the study period (*p* = 0.93 and *p* = 0.32, respectively). Although there was little difference in rates of change, rural counties generally had higher bladder cancer mortality rates compared to urban counterparts at 5.10 cases per 100,000 versus 4.70 cases per 100,000, respectively (Figure 2C).

For kidney cancer, the majority of new cases were diagnosed in urban counties (72.3%). Age-adjusted kidney cancer incidence significantly increased over time in both rural counties (65.8% increase, AAPC 11.2, *p* < 0.01) and in urban counties (59.0% increase, AAPC 9.3, *p* = 0.01, Figure 1D). Age-adjusted kidney cancer mortality in urban counties decreased by 18.9% from 4.17 to 3.38 cases per 100,000 (AAPC −6.9% *p* = 0.03), while mortality in rural counties remained stable at 3.85 cases per 100,000 (*p* = 0.16, Figure 2D).

## 4. Discussion

In this study, spanning nearly three decades, of the Pennsylvania Cancer Registry, we found decreases in overall GU cancer and prostate cancer incidence in both rural and urban counties; however, in rural counties, bladder cancer incidence remained stable and kidney cancer incidence increased. We also found that overall GU cancer and prostate cancer mortality decreased in rural and urban counties; however, bladder cancer and kidney cancer mortality remained stable. The decreases in overall GU cancer incidence and mortality are likely driven by improvements in prostate cancer, which was the majority of new GU cancer diagnoses. In contrast, bladder cancer and kidney cancer incidence and mortality remained stable or worsened over the study period. In a more general context, within the Commonwealth of Pennsylvania, there were 77,139 new cancer cases diagnosed in 2021 (age-adjusted incidence rate 437.6 per 100,000 people) and 27,637 deaths attributed to cancer (age-adjusted mortality rate 149.9 per 100,000 people) [10].

Our findings in Pennsylvania track national trends in GU cancers. A recent study of national urban–rural cancer mortality using over 20 years of National Center for Health Statistics data found that bladder cancer mortality was mostly unchanged across both urban and rural counties (AAPC −0.61 versus 0.08) while there were large decreases in prostate cancer mortality in both urban and rural counties (AAPC −2.48 versus −2.54). Kidney cancer mortality decreased some in urban counties while remaining mostly flat in rural counties (AAPC −1.14 versus −0.33) [11].

Our finding that prostate cancer incidence has decreased in Pennsylvania corroborates a prior study of the Pennsylvania Cancer Registry of data from 2009 to 2015 in which the authors noted an overall decrease from 416 to 304 per 100,000 men [12]. Additionally, the authors examined the receipt of treatment for prostate cancer by urban–rural status and disease severity. At all levels of prostate cancer severity, rural men were less likely to receive any treatment (adjusted odds ratio 0.73, 95% CI 0.65–0.83). There were no differences in the proportions receiving radiation therapy across disease severity and urban–rural status (urban 33% versus large town 32% versus rural 32%). Increasing age was also associated with a lower likelihood of being treated for prostate cancer (adjusted odds ratio 0.78 for men aged 55–59 years versus adjusted odds ratio 0.28 for men > 74 years). Rural areas tend to have a larger proportion of older adults, and age could drive the lack of overall prostate cancer treatment in rural Pennsylvania. In a study that utilized the National Surveillance, Epidemiology, and End Results database (SEER), both rural and urban counties had similar cancer-specific mortality (CSM) and minimal differences in prostate cancer stage at presentation and treatment rates [13]. The authors report no significant difference in the 10-year CSM rates (40.1% versus 43.8%, *p* = 0.4) for rural areas versus urban areas. However, other cause mortality was higher in prostate cancer patients in rural areas when compared to urban clusters and areas. The authors reported 10-year other cause-mortality rates for rural areas versus urban clusters versus urban areas as 27.2%, 23.7%, and 18.9%, respectively (*p* < 0.001). One potential reason for these differences in other cause mortality is rural–urban disparity in chronic diseases. Health outcomes for chronic diseases are worse in rural areas which may contribute to deaths from other causes in rural prostate cancer survivors [14]. Chronic disease mortality in rural GU cancer survivors is a priority area for future research and interventions to reduce cancer survivorship disparities.

In Pennsylvania, we found that rural counties had higher rates of bladder cancer mortality compared to urban counties, although cancer mortality and incidence rates remained stable over the study period. Our findings are similar to a SEER study of bladder cancer in which rural counties in Utah had a 5-year relative bladder cancer survival that was 5.2% lower than urban counties and a 10% increased risk of death [15]. Bladder cancer is one of the most expensive cancers to treat from diagnosis to death with an estimated lifetime cost of between USD 9600 and USD 18,700 due to the ongoing need for frequent surveillance procedures (cystoscopy), surgeries, and bladder instillations [16,17]. This high cost burden coupled with social vulnerabilities present in many rural counties and increased travel distances introduces treatment barriers that may be associated with higher bladder cancer mortality in rural Pennsylvania counties. A geographical study of the U.S. Medicare database found that only 13% of the U.S. population lives within 30 min driving time of a urologist and that, in large areas of the Western frontier region, Medicare beneficiaries travel > 60 min to access urologic care [18].

We found that kidney cancer incidence increased in rural and urban Pennsylvania counties, while mortality decreased in urban counties and remained stable in rural. Kidney cancer incidence overall has increased, likely due to increased detection and risk factors such as rising obesity rates [19]. Rural residents may be more susceptible to kidney cancer as kidney cancer-related risk factors such as obesity, as well as risky health behaviors (e.g., smoking), occur at higher rates in rural areas [20,21]. Smoking and obesity are key risk factors for bladder cancer and kidney cancer, respectively.

In addition, stable kidney cancer mortality in rural counties and decreasing mortality in urban environments may be explained by low urologist density in rural counties [22]. Urologists are the first line for diagnosis and treatment of GU cancers; however, only approximately 10% of urologists practice in rural counties [23]. Urologists in rural areas are older (60 years) than urban urologists and have more years in practice, and the gap is widening as younger urologists increasingly choose urban practices [24]. Impending rural urologist retirements will compound the ongoing shortage of rural urologists and have the potential to worsen GU cancer mortality rates.

In consideration of the evidence that emerged from our study, interventions to address rural–urban GU cancer disparities may include health policy that increases urologic care access. For example, the COVID-19 pandemic expanded telehealth access and reimbursement for clinicians with emergency legislation. Expanding telehealth to rural health systems and communities reduces barriers to urologic access by minimizing travel burdens and resulting out-of-pocket costs. New legislation efforts in the U.S. government have targeted making the temporary COVID-19 emergency telehealth expansions permanent. Additional legislative efforts have focused on increasing the number of urologists working in rural areas.

Our findings must be considered within the setting of some limitations. We utilized the Center for Rural Pennsylvania’s 2010 rural–urban county classification and applied it across all years in our dataset. It is possible that certain counties may have shifted from rural to urban or vice versa over the 30-year study period. Due to limitations of the joinpoint regression program, we were unable to adjust for changes in rural–urban classification over time. The Pennsylvania Cancer Registry database lacked more detailed information on individual patient and tumor characteristics, and we were unable to adjust for variables such as insurance status or cancer stage.

To conclude, in this 30-year review of the Pennsylvania Cancer Registry, overall GU cancer incidence and mortality decreased across both rural and urban Pennsylvania counties. For prostate cancer, both incidence and mortality decreased. Bladder cancer incidence and mortality remained stable over time. Kidney cancer incidence increased across all counties; however, mortality was stable in rural counties. In the future, these findings will help us to understand the burden of common GU cancers in rural areas and may focus efforts to improve care and outcomes for rural individuals with GU cancers.

## Figures and Tables

**Table 1:** New genitourinary cancer diagnoses by year and rural/urban county in the Pennsylvania cancer registry.

	Years					
GU Cancer Type	1990–1994	1995–1999	2000–2004	2015–2019	2011–2014	2015–2019
**All, n (%)**						
Rural	19,379 (27.4%)	21,212 (28.5%)	22,912 (28.7%)	24,233 (29.4%)	21,528 (28.2%)	22,463 (28.4%)
Urban	51,132 (72.5%)	53,252 (71.5%)	57,013 (71.3%)	58,248 (70.6%)	54,929 (71.8%)	56,599 (71.6%)
**Prostate, n (%)**						
Rural	12,968 (27.3%)	14,038 (28.8%)	14,780 (28.8%)	15,039 (29.4%)	12,035 (27.9%)	12,309 (27.3%)
Urban	34,479 (72.7%)	34,724 (71.2%)	36,461 (71.2%)	36,156 (70.6%)	31,111 (72.1%)	32,787 (72.7%)
**Bladder, n (%)**						
Rural	4516 (28.2%)	4988 (28.7%)	5394 (29.3%)	5773 (30.3%)	5891 (29.1%)	5987 (30.3%)
Urban	11,513 (71.8%)	12,400 (71.3%)	13,019 (70.7%)	13,282 (69.7%)	14,369 (70.9%)	13,794 (69.7%)
**Kidney, n (%)**						
Rural	1895 (26.9%)	2186 (26.3%)	2738 (26.7%)	3421 (28%)	3612 (27.7%)	4167 (29.4%)
Urban	5140 (73.1%)	6128 (73.7%)	7533 (73.3%)	8810 (72%)	9449 (72.3%)	10,018 (70.6%)

## Data Availability

The original data presented in this study are openly available in the Pennsylvania Cancer Registry’s Enterprise Data Dissemination Information Exchange (EDDIE) at https://www.pa.gov/en/agencies/health/health-statistics/eddie-about.html (accessed on 17 December 2024).
